# Optimizing Melamine Resin Microspheres with Excess Formaldehyde for the SERS Substrate

**DOI:** 10.3390/nano7090263

**Published:** 2017-09-06

**Authors:** Lu Shen, Junfu Zhu, Yuqing Guo, Zhirong Zhu, Xiaogang Wang, Zhixian Hao

**Affiliations:** Shanghai Key Laboratory of Chemical Assessment and Sustainability, School of Chemical Science and Engineering, Tongji University, Shanghai 200092, China; shenlu@tongji.edu.cn (L.S.); zhujunfu0@126.com (J.Z.); gyq051212@163.com (Y.G.); zhuzhirong@tongji.edu.cn (Z.Z.); xgwang@tongji.edu.cn (X.W.)

**Keywords:** excess monomer, melamine resin microspheres, SERS substrate, environmental contaminants

## Abstract

Influence of the excess monomer within the synthetic reaction solution of melamine resin microspheres (MFMSs) on the surface-enhanced Raman spectroscopy (SERS) enhancement from Rhodamine 6G (R6G) was investigated, where the R6G was adsorbed on the silver nanoparticles (AgNPs) that were loaded on the MFMSs. Surface characteristics of the MFMSs were modified by the excess monomer (i.e., the excessive melamine or formaldehyde) through its terminal overreaction, which can be simply controlled by some of the synthetic reaction conditions, thus further allowing us to optimize the assembly of the loaded AgNPs for the SERS detection. These SERS substrates incorporating the optimized MFMSs with the excess formaldehyde can also be used for tracing analyses of more environmental and food contaminants.

## 1. Introduction

A linear polymerization reaction between two co-monomers A and B is ideal if its polymer molecules could be strictly expressed as B-[A-B]*_n_*-A while the “*n*”, degree of polymerization, approaches infinity. Nevertheless, besides remaining in the reaction solution, the excessive one within the two co-monomers can also bond at both ends of the structure, and thereby serve as an inhibitor to adjust the polymerization degree, or even as a functional group located at both ends. From the perspective of geometry, if a copolymer molecule regularly grows up along one-, two-, or three-dimensional directions, it is possible for the involved excess co-monomer to be bonded at its two ends, outside its planar contour or on its spherical surface. Furthermore, some complicated structures may appear in the copolymer product if it precipitates out of the reaction solution. This type of precipitation is not only pushed forward by its main polymerization reaction, but also by some of the cross-linking or/and crystallization processes, e.g., lamellar crystallization caused by hydrogen bonding among different linear molecules [1,2,3,4], which are always variable with the reaction time or the molar ratio between its two co-monomers. Therefore, it should be very important to use these copolymerization reaction conditions to modify the polymer precipitates so as to obtain some optimized functional materials.

Melamine-formaldehyde resin (MF) from the polycondensation reaction is well-known due to its wide application in the wood and papermaking industries, as well as dinnerware manufacturing [5,6]. Wherein, the MF microspheres can precipitate out of the reaction solution under some special conditions [7,8]. MFMSs were introduced into the SERS substrate for the trace analysis of tetramethylthiuram disulfide, uric acid, and other analytes [9,10,11], in which the MFMSs were synthesized by successive catalysis of alkali and acid at a constant formaldehyde/melamine (F:M) molar ratio and the deposition of AgNPs on MF microspheres was carried out by reduction of AgNO_3_ using butylamine as the reducing agent. In addition to the complexity of the procedure, what role the F:M molar ratio played in the MFMS synthesis and, furthermore, in the performance of SERS substrates is still unknown.

SERS hot spots between metal nanoparticles can be created by some bottom-up methods [12,13], which were shown to be a successful strategy by controlling the distance between the metal nanoparticles using some linkers, e.g., hexamethylenediamine [14], 1,4-benzenedithiol [15], and some special copolymers [16,17,18]. This should be further evidence for the SERS hot spots that SERS enhancement on the silver nanoparticles increases with the number of their aggregate-dimensions controlled by some organic molecules increasing from one to two and then to three [19]. Introducing polymeric carriers into the SERS substrate to enhance the detection sensitivity has become a common practice [9,10,11,20,21,22,23]. This method depends on the existence of numerous functional groups within the polymer, which are available to the three-dimensional aggregation of the metal nanoparticles. However, it is troublesome that the variable synthetic conditions frequently result in some different characteristics to the polymers, especially to some copolymers [9,10,11,20,22,24], thus, further influencing the properties of the final SERS substrate [9,10,11,20,22]. Obviously, how to classify or optimize the polymer involved in the fabrication of SERS substrates is vital.

In our last paper, the types of urea-formaldehyde resin microspheres were investigated where the role of excess monomer in the synthetic process was highlighted [24]. Indeed, it is a common phenomenon that the copolymer resin will present at least two types of characteristics, which correspond to its modification with the excess one of the two co-monomers. In this paper, MFMSs were synthesized to simply load AgNPs from the colloidal solution for the SERS detection. The excess monomer bonded on the MFMSs was found further playing a crucial role in their SERS substrate, so as to impact on the final SERS detection. The SERS substrate optimized with the excess formaldehyde can be used for the trace analyses of a set of organic pollutant molecules containing mercapto, thiocarbonyl, or quaternary amino groups.

## 2. Results and Discussion

**Figure 1:**
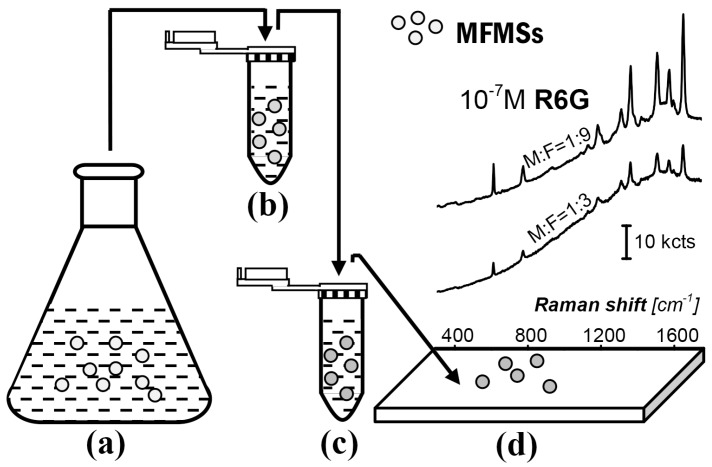
A route for the fabrication and use of SERS substrate AgNP/MFMSs. (**a**) Synthesis reaction with melamine and formaldehyde for MFMSs; (**b**) adsorption of AgNPs on the MFMSs in silver colloidal solution; (**c**) incubation of the AgNP/MFMSs sample in the R6G solution; and (**d**) interrogation of the SERS substrate sample with Raman spectroscopy.

A simple route for the fabrication and use of the SERS substrate, AgNP/MFMSs, is provided in Figure 1. MFMSs were firstly synthesized with melamine and formaldehyde by catalysis of acetic acid (Figure 1a). They were transferred into a silver colloidal solution to adsorb AgNPs (Figure 1b). The AgNP/MFMSs were then transferred into R6G solution to adsorb R6G (Figure 1c). Finally the incubated AgNP/MFMSs sample was put on a glass slide to interrogate the R6G (Figure 1d).

AgNPs were loaded on MFMSs within a 2 mL centrifuge tube, followed by an incubation step in a 10^−7^ M R6G solution. The colloidal solution was firstly centrifuged at 2000 rpm before use to remove the large AgNPs within itself. The synthesis of the MFMSs was implemented at pH 4.0 and 65 °C with a constant amount of melamine, and either the reaction time or the initial formaldehyde amount was preset as a variable, which further incorporated into the AgNP/MFMSs samples, to investigate their influence on the final SERS performance. Size of the AgNPs was ~120 nm and that of the MFMSs varied from 12.4 ± 2.21 to 5.8 ± 0.47 µm with the F:M molar ratio increasing from 1:1 to 9:1 (see Table S1, which provided in the Supplementary Material). The zeta potentials of MF_1_ and MF_7_ were + 18.7 mV and + 36.6 mV, respectively. After an ultrasonic oscillation step in AgNP colloidal solution for 5 min, the MFMSs were allowed to remain in the solution for 1 h to adsorb the AgNPs, and then collected at a centrifugation rate of 2000 rpm. It was verified that the AgNPs on the MFMSs has an fcc structure, as shown in the x-ray diffraction (XRD) spectra in Figure S1.

**Figure 2:**
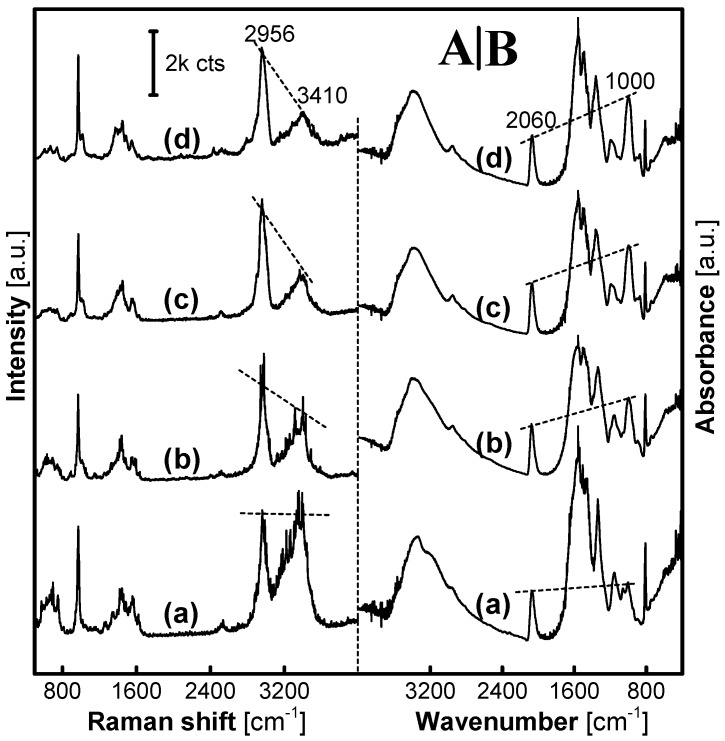
Raman and IR spectra of a set of MFMSs synthesized in 3 h. (**A**) Raman spectra of MF_1_ (**a**), MF_3_ (**b**), MF_7_ (**c**) and MF_9_ (**d**); (**B**) IR spectra of MF_1_ (**a**), MF_3_ (**b**), MF_7_ (**c**), and MF_9_ (**d**). A laser power of 20 mW was chosen in the collection of Raman spectra of MFMSs.

Figure 2 gives Raman and Infrared (IR) spectra of a set of MFMS samples. With the F:M molar ratio increased from 1:1 to 9:1, a Raman scattering peak at 2900 cm^−1^ that could be assigned to the stretching vibration of C–H in the groups –CH_2_– or/and –CH_2_OH was enhanced along with a gradual decline of another one around 3400 cm^−1^, which was attributed to the vibration of N–H and/or O–H in the –NH–, –NH_2_ and/or –CH_2_OH, as shown in Figure 2A. IR spectra, as shown in Figure 2B, used Pb(SCN)_2_ (50 wt%) as an internal standard with its characteristic peak at 2060 cm^−1^ to evaluate the change of the peak intensity at 1000 cm^−1^. The adsorption peak at 1000 cm^−1^ was assigned to the C–O vibration that was associated with the existence of –NHCH_2_OH groups in the MFMSs. Herein, with the F:M molar ratio increasing from 1:1 to 9:1, the intensity of the peak at 1000 cm^−1^ increased. Therefore, it is an important conclusion from the results of Raman and IR analyses that the number of the –NHCH_2_OH groups in the MFMSs increases with the F:M molar ratio increasing from 1:1 to 9:1 in the synthesis of MFMSs. Herein, it is also possible for X-ray diffraction and particle size, as well as the yield of resin materials to change gradually, e.g., as reported in the synthesis of urea-formaldehyde resin microspheres [24,25].

**Figure 3:**
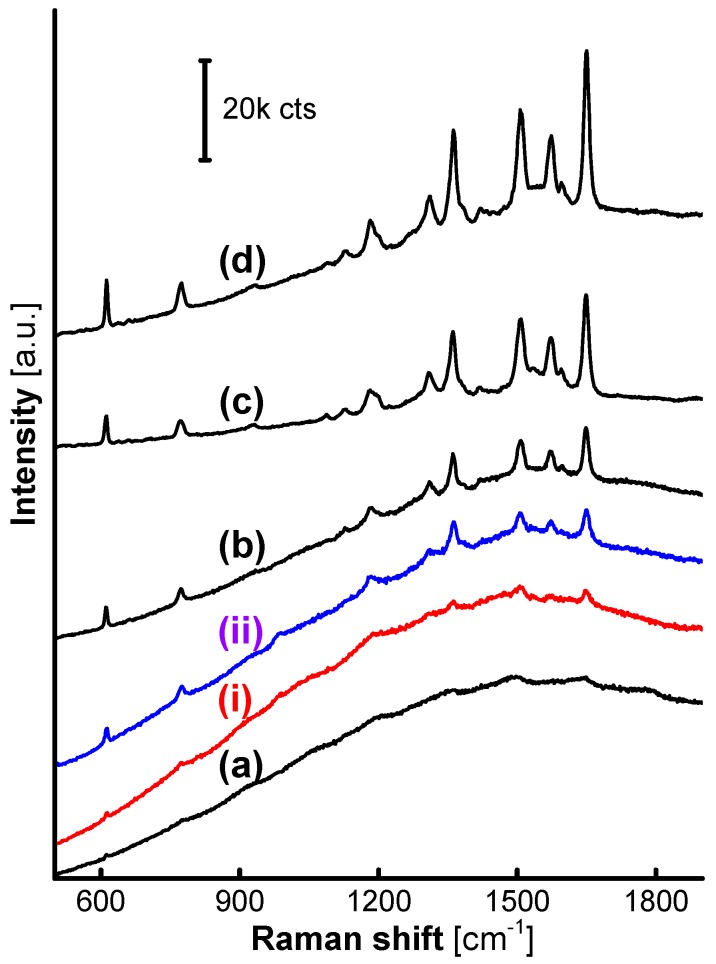
SERS spectra from R6G adsorbed on the AgNPs loaded on MF_1_ (**a**), MF_1.5_ (**i**), MF_2_ (**ii**), MF_3_ (**b**), MF_7_ (**c**), and MF_9_ (**d**). Only 1% of 20 mW was adopted in the collection of the SERS spectra.

These trends in the structure of the MFMSs are further transferred into a gradually-increasing SERS performance from R6G loaded on the AgNPs. As presented from Figure 3a–d, the intensity of SERS signals from R6G on the loaded AgNPs were strikingly enhanced with the F:M molar ratio increasing from 1:1 to 9:1, while the Raman signals of the MFMSs themselves were faint, and almost only an intense fluorescence background was observed on the AgNP/MF_1_MSs (see Figure 3a). Only one percent of the 20 mW laser power was adopted in the detection of SERS spectra, reflecting the superiority of SERS analysis on the AgNP/MFMSs (herein “AgNP” and “MF” in the expression AgNP/MF_a_MSs denote AgNPs and MFMSs, respectively, and the subscript “a” is the molar ratio between formaldehyde and melamine preset in the MF polycondensation reaction).

Obviously, there must be a specific value of the F:M molar ratio between 1:1 and 3:1 that can demarcate the characteristics of the MFMS surfaces and thereby divide the loaded AgNPs into active and inactive ones based on the SERS performance. Two MFMS samples were further synthesized at the F:M molar ratio of 1.5:1 (MF_1.5_) and 2:1 (MF_2_), respectively, to evaluate their SERS performance. SERS signals originating from R6G on the AgNP/MF_2_MSs (ii) emerged clearly, though there existed an intense fluorescence background in each one of the spectra collected on AgNP/MF_2_MSs (ii), AgNP/MF_1.5_MSs (i) and AgNP/MF_1_MSs (a) as shown in Figure 3. Although the exact structure of the MF_1.5_ is still waiting for verification, it is evident from the SERS effectiveness that the initial F:M molar ratio of 1.5:1 in the MFMS synthesis reaction is a valuable reference, on which it is reasonable for the surface characteristics of the MFMSs to be demarcated by the excess formaldehyde [24] or melamine.

**Figure 4:**
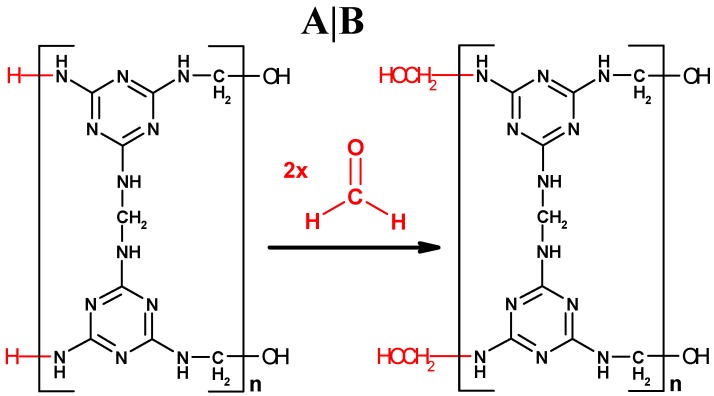
The terminal overreaction of a linear MF resin molecule. (**A**) An linear molecule extended from an ideal polymerized unit C_9_N_12_H_12_, posed from the F:M molar ratio of 1.5:1; and (**B**) the linear molecule with –NH_2_ groups further bonded by the excessive formaldehyde.

Assuming that the MF polycondensation reaction strictly complies with the F:M molar ratio of 1.5:1, an ideal structure in linear molecular form is argued as Figure 4A, based on its ideal polymerized unit C_9_N_12_H_12_ within Figure 4A. In the structure Figure 4A, all the –NH_2_ groups from the reactant melamine are changed into –NHCH_2_– after the polycondensation reaction, except the two remaining at the left end of the resin molecule.

We would like to emphasize the “overreaction” between the –NH_2_ groups in the MF resin molecule and the excess formaldehyde remaining in the reaction solution. If the MF polycondensation reaction occurs in the solution with more amount of formaldehyde than it needs, the formaldehyde will excessively bond at the ends of MF resin molecules after the melamine in the reaction solution is exhausted. The two –NH_2_ groups remained at the left end of the linear molecular structure will be further consumed by their overreaction with the excess formaldehyde, as shown from (A) to (B) in Figure 4. This process is similar if a resin molecular structure grows in two- or three-dimensions, where some of the excess monomer in the reaction solution will be bonded at more or all terminal sites as the polycondensation reaction is over. The terminal overreaction between the –NH_2_ groups in a two-dimensional MF resin molecule and the excess formaldehyde remaining in the reaction solution is further illustrated in Figure S2.

Although a MFMS is not a single resin macromolecule grown in three-dimensions, the “terminal overreaction” could obviously be used to understand its growth process. The MFMS will have to be exposed in the polycondensation mother liquor and it is thereby possible for the excessive reactant in the solution to be bonded enough on the surface of it. When the F:M molar ratio is more than its ideal F:M molar ratio of 1.5:1, the surface of the MFMSs with bonded –NH_2_ groups will finally be transferred into the one that was enriched with –NHCH_2_OH, because there is excessive formaldehyde in the reaction solution throughout the synthesis reaction.

**Figure 5:**
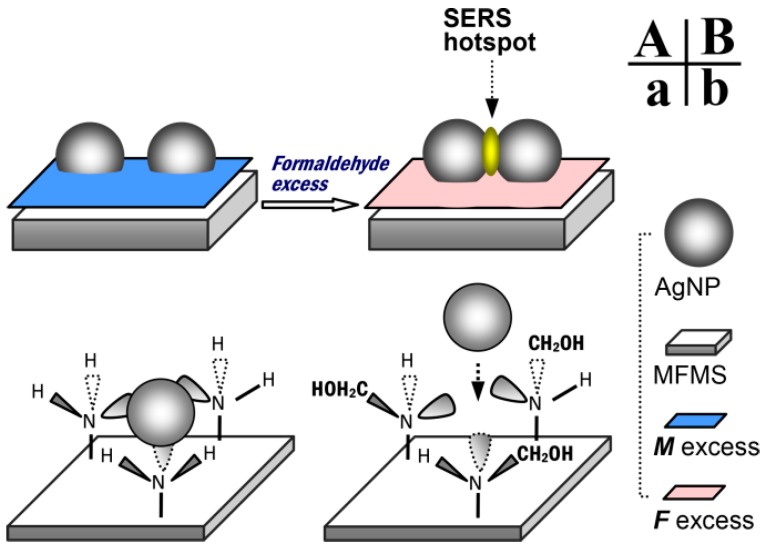
A possible explanation for the formation of SERS hot spot on the AgNP/MFMSs. (**A**) and (**a**): Strong complexing of –NH– in –NH_2_ group to AgNP on the MFMS with melamine excess, the AgNPs were anchored on the surface of the MFMS; (**B**) and (**b**): Weak complexing of –NH– in –NHCH_2_OH group, with an additional steric hindrance from the –CH_2_OH to AgNP on the MFMS with formaldehyde excess, thus allowing the two AgNPs to sufficiently interact with each other to form a SERS hot spot [12,13].

Figure 5 gives a possible explanation for the formation of SERS hot spots on the AgNP/MFMSs. On the surface of MFMS with formaldehyde excess, the –NH– in the –NHCH_2_OH group has a weak capability to complex with AgNP, thereby allowing two AgNPs to interact with each other sufficiently to form an effective SERS hot spot, as shown in Figure 5B,b.

**Figure 6:**
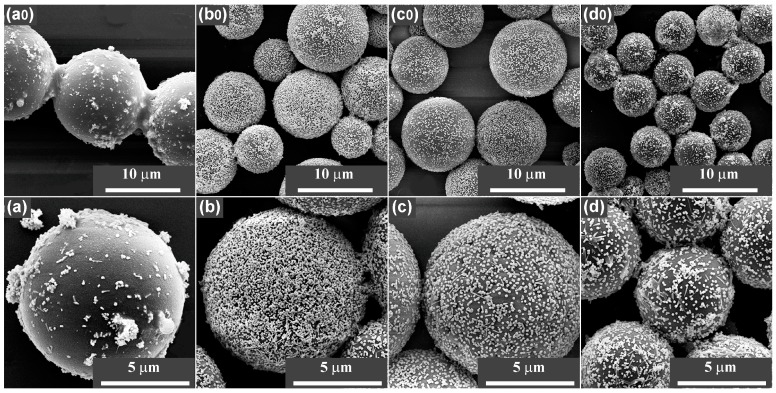
SEM images of AgNPs on MFMSs synthesized with a set of F:M molar ratios in 3 h. (**a**,**a0**) MF_1_; (**b**,**b0**) MF_3_; (**c**,**c0**) MF_7_; and (**d**,**d0**) MF_9_. In the case of AgNP/MF_1_MSs (**a**,**a0**), the AgNPs aggregation was severe, and is also shown in additional SEM images provided in Figure S3.

Dispersion characteristics of the AgNPs on the MFMSs are shown in their scanning electron microscope (SEM) images (Figure 6). The AgNPs had been perfectly dispersed on the samples of AgNP/MF_3_MSs (Figure 6(b,b0)), AgNP/MF_7_MSs (Figure 6(c,c0)), and AgNP/MF_9_MSs (Figure 6(d,d0)), while they were severely aggregated on AgNP/MF_1_MSs (also see Figure S3). The density of the AgNPs on the surface AgNP/MF_3_MSs seem to be the highest, indeed the contents of AgNPs in all the AgNP/MFMSs samples were roughly consistent (5.02–6.42%, as shown in Table S2). These results demonstrated that the SERS performance of the substrate is independent of the silver loading efficiency, but strongly related to the assembly of AgNPs on the surface of MFMSs. The surface with excess formaldehyde favors the assembly of AgNPs and, thus, the formation of SERS hot spots.

**Figure 7:**
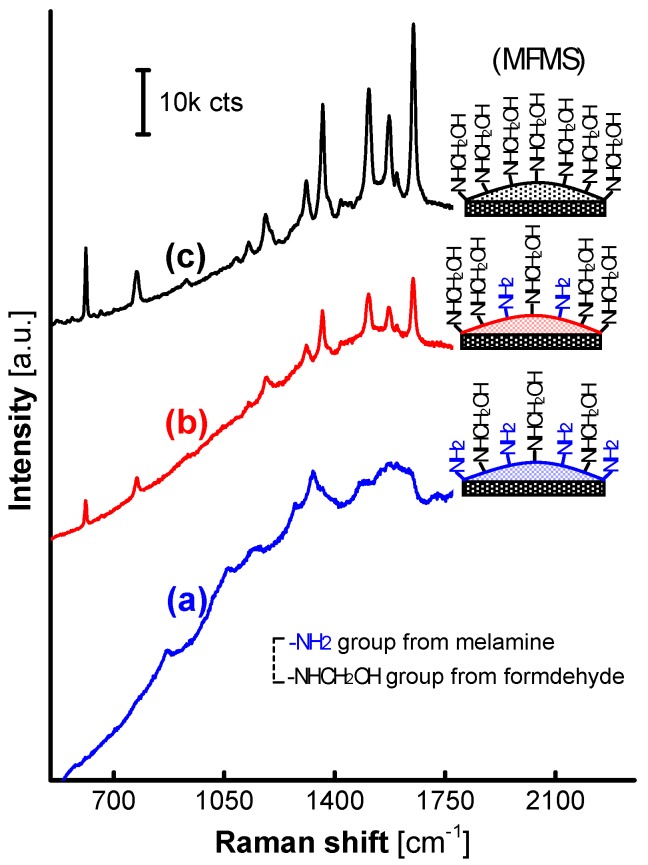
SERS spectra from R6G on AgNPs loaded on MF_3_ synthesized in a set of reaction time. (**a**) 20 min; (**b**) 3 h; and (**c**) 8 h.

The reaction time in the MFMS synthesis is another important variable. The excess co-monomer will remain in the solution following the exhaustion of the other inadequate one and be bonded on the surface of MFMSs to the maximum extent at the end of the synthesis reaction. With the reaction time increased from 20 min to 8 h, the fluorescence background in the SERS spectra on AgNP/MF_3_MSs declined, but the SERS signals gained intensity gradually, as shown in Figure 7. Herein, the amount of formaldehyde bonded on the MFMS surface was speculated to increase with the exhaustion of the melamine in the reaction solution, and the AgNPs that were thereby activated on the surface modified gradually until completion of the reaction.

**Figure 8:**
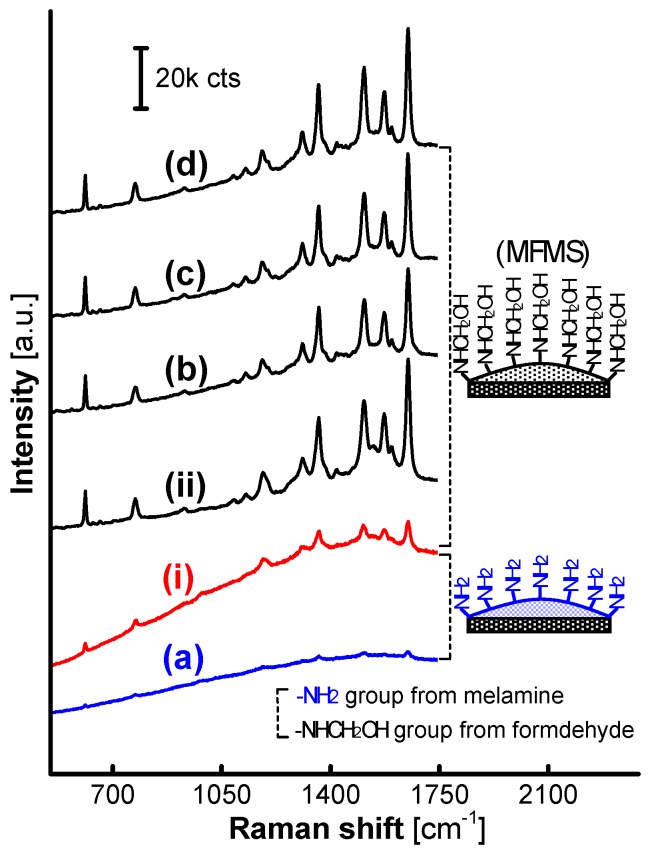
SERS spectra from R6G on AgNPs loaded on MFMSs synthesized with a set of F:M molar ratios in 8 h. (**a**) MF_1_; (**i**) MF_1.5_; (**ii**) MF_2_; (**b**) MF_3_; (**c**) MF_7_; and (**d**) MF_9_.

The specific demarcation F:M molar ratio, 1.5:1, was again confirmed by the SERS spectra on AgNPs loaded on the MFMSs that were synthesized in 8 h, as shown in Figure 8. Herein, the SERS signals all gained striking intensity if the incorporated MFMSs synthesized at the F:M molar ratio more than 1.5:1, except those faint on MF_1.5_ (i) and no SERS signals on MF_1_ (a) that were obviously inactivated by the excess melamine bonded on the surface.

Oligomers incorporated in MFMSs were suspected to be involved in the aggregation of the AgNPs, to thereby influence the SERS enhancement. If they did, they should be dissolvable to a certain extent in water because of the existence of hydrophilic melamine and/or formaldehyde bonded to them, and might work as linkers involved in the construction of SERS hot spots between the AgNPs [14,15,16,17,18]. To evaluate the influence of MF oilgomers on the SERS enhancement, MFMSs were dispersed into water under ultrasonic oscillation, impregnated for 12 h, and then removed via centrifugation. Herein, the AgNPs, separated via centrifugation between 2000 and 6000 rpm, were treated by the impregnating solution and then used directly for the SERS detection following its R6G incubation.

**Figure 9:**
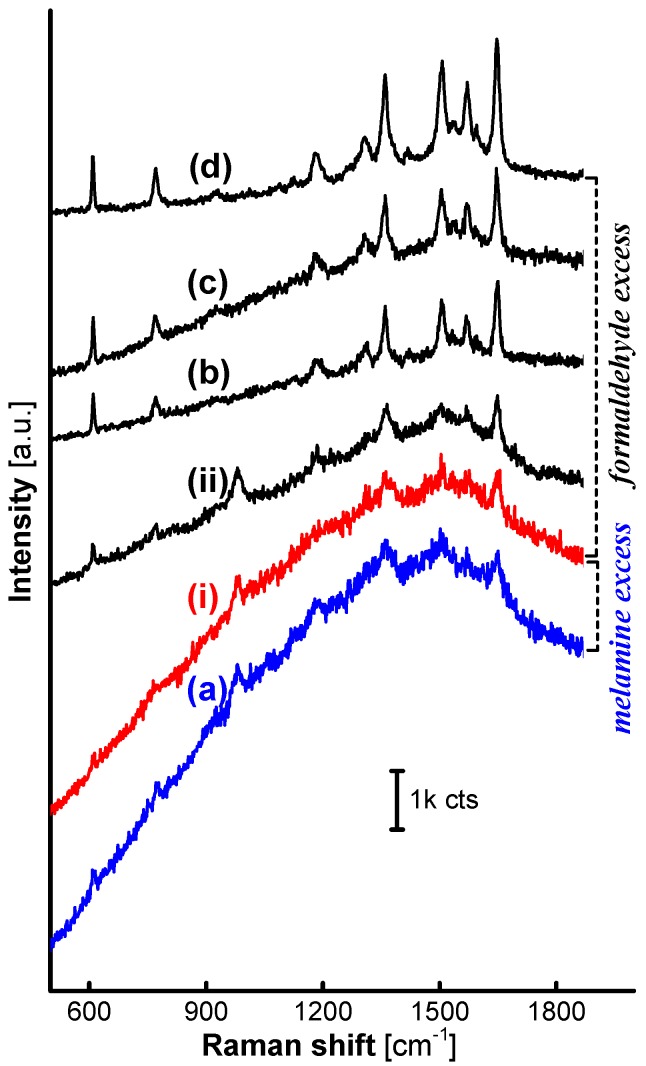
SERS spectra from R6G on the AgNPs treated with the solutions, which had impregnated the MFMSs synthesized with a set of F:M molar ratios in 3 h. (**a**) MF_1_; (**i**) MF_1.5_; (**ii**) MF_2_; (**b**) MF_3_; (**c**) MF_7_; and (**d**) MF_9_.

With the F:M molar ratio increased, the AgNPs gave SERS signals following a trend of the intensity just similar to that observed on the AgNP/MFMSs samples (compare Figure 9 with Figure 3), but the intensity of the SERS signals was only about one-twentieth the value observed on the AgNP/MFMSs. Obviously, the oligomers impregnated out at ambient temperature are effective in the SERS detection, but only play a minor role in the presence of MFMSs. These results, again, verified the importance of excess formaldehyde based on the small molecular level, while providing additional evidence for the complexing rationale from the –NH– groups to AgNPs, as shown in Figure 5.

SERS spectra measured on AgNP/MFMSs incubated with a set of R6G concentrations are shown in Figure S4. With the concentration decreased from 10^−7^ to 10^−13^ M, the SERS signal from R6G gradually declined to a R6G detection limit of 10^−13^ M. The SERS enhancement factor (EF) is estimated to be about 1.24 × 10^8^ (Figure S5) [26,27]. The relative standard deviation (RSD) of the SERS peak intensity in detection of 10^−7^ M R6G was found to be less than 15%, as shown in Figure S6 and Table S3. Therefore, AgNP/MFMSs can be used as a simple and effective SERS substrate.

**Figure 10:**
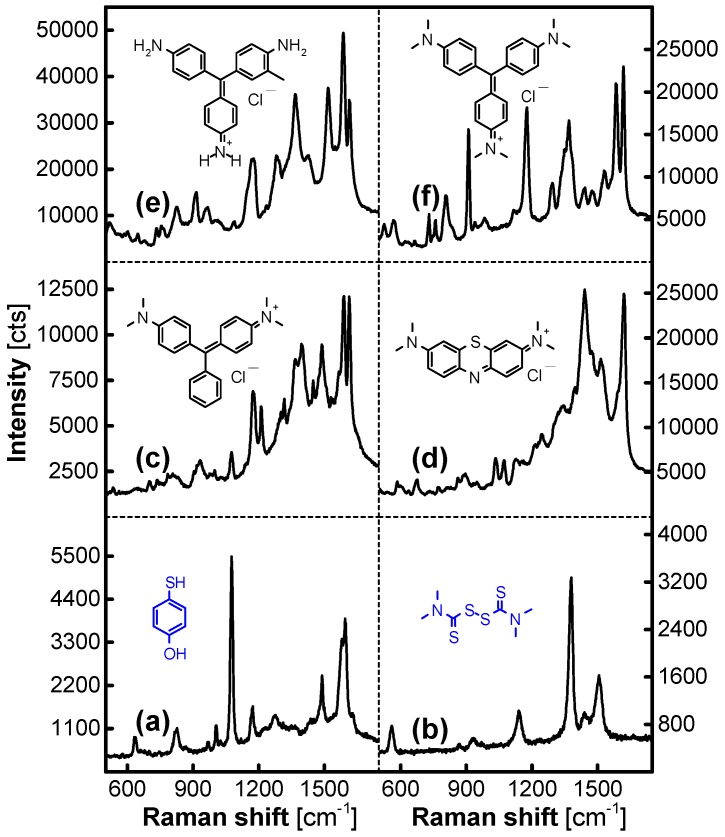
SERS spectra from a set of organic analytes (10^−5^ M) on AgNPs loaded on MF_3_ synthesized in 8 h. (**a**) *p*-hydroxythiophenol (CAS: 637-89-8; pharmaceutical and dye intermediate); (**b**) tetramethylthiuram disulfide (CAS: 137-26-8; disinfectant, insecticide, and vulcanization accelerator in rubbers); (**c**) malachite green (CAS: 2437-29-8; dye and toxic antiseptic for aquaculture); (**d**) methylene blue (CAS: 7220-79-3; dye and drug); (**e**) basic violet 14 (CAS: 632-99-5; dye); and (**f**) crystal violet (CAS: 548-62-9; disinfectant and toxic antiseptic for aquaculture).

AgNP/MFMSs as a SERS substrate can be used to detect organic molecules with mercapto or thiocarbonyl groups that can covalently bond with Ag atoms, such as *p*-hydroxythiophenol and tetramethylthiuram disulfide in ethanol solution, as shown in Figure 10a,b. It is competent for analyzing organic amine salts in aqueous solution, e.g., trace amounts of Rhodamine 6G, malachite green, methylene blue, basic violet 14, and crystal violet, as shown in Figure 10c–f, which is significant in the detection of environmental and food contaminants. We would like to explain this feature by the similar compatibility between the organic amino molecules and the MFMSs, and it should be helpful for the organic amino molecules to be collected and enriched by the MFMSs in the incubation step.

## 3. Methods

### 3.1. Syntheses of MFMSs and AgNP Colloidal Solution

The MFMSs was synthesized by the polycondensation-precipitation reaction between melamine and formaldehyde [7,8] catalyzed by acetic acid. Acetic acid solution (pH 4.0, 90 mL), melamine (2.5 g), and a variable amount of formaldehyde solution (38 wt %) were mixed in a conical flask, and the mixture was heated to, and maintained at, 65 °C with electromagnetic stirring until the melamine was dissolved. When the reaction mixture became cloudy [24], it was kept at 65 °C for a further preset time, allowing the initial nuclei of the MFMSs to form. When the mixture was cooled, the MFMSs were collected by filtration through a Buchner funnel and washed with water and ethanol three times, respectively. Following an air-dry step overnight at ambient temperature, the MFMS sample was further dried in an oven at 50 °C for 5 h and then kept in a desiccator for use. Herein, either the reaction time following the cloudy state or the F:M molar ratio adopted in the synthesis was preset as a variable, which will be incorporated into the SERS substrate, to investigate the final SERS performance.

AgNP colloidal solution was synthesized following a standard procedure [22,28]. As soon as 5 mL of Na_3_C_6_H_5_O_7_ solution was added, a boiling AgNO_3_ solution (95 mL) in a glass beaker was sealed with a piece of plastic paper and then kept boiling for an additional 30 min. The initial concentrations of the Na_3_C_6_H_5_O_7_ and AgNO_3_ solutions in the reaction were equal to 3.0 × 10^−3^ and 2.0 × 10^−3^ M, respectively. The water, used in the syntheses of the AgNP colloidal solution and the fabrication of subsequent SERS substrate, had an electrical conductivity of 1.37 µS/cm. All chemicals were of analytical grade from Sinopharm Chemical Reagent Co., Ltd. (Shanghai, China), and were used directly without further purification.

### 3.2. Fabrication and Incubation of the SERS Substrate AgNP/MFMSs

The fabrication of AgNP/MFMSs was implemented in situ in a 2 mL centrifuge tube prior to use. AgNPs in 2 mL colloidal solution were firstly separated through two sequential centrifugation steps at 2000 and 6000 rpm for 5 min, respectively, where the precipitate from the step at 2000 rpm and the supernatant from the one at 6000 rpm were discarded, and then re-dispersed into water to the initial volume. A 5.0 mg MFMS sample was finally impregnated in the AgNP solution for 60 min and separated at 2000 rpm to complete the AgNP loading procedure.

Incubation of the AgNP/MFMSs using R6G as a target analyte was still carried out in situ in the centrifuge tube. The SERS substrate was dispersed and kept impregnated in 2 mL R6G solution at a concentration of 10^−7^ M for 2 h. After separating at 2000 rpm, a small amount of the sample was transferred onto a glass slide, which had been pretreated with ethanol, and dried in a desiccator for 12 h for Raman spectroscopy interrogation. Some other analytes in solutions at 10^−5^ M, including *p*-hydroxythiophenol and tetramethylthiuram disulfide in ethanol, and malachite green, methylene blue, basic violet 14, and crystal violet in water, were finally used in the incubation of the optimized substrate to verify their SERS performance.

### 3.3. Characterization of the SERS Substrate

Raman spectrum was measured on a confocal microscope Raman spectrometer (Invia Reflex Renishaw, Renishaw Apply Innovation, Gloucestershire, UK) with a 514.5 nm laser in excitation through a 50× microscope objective. A 20-mW laser was chosen in the Raman spectrum detector and only 1% of its power was adopted in the SERS investigation. The accumulation time was 10 s. The IR spectra of the samples were analyzed with a Fourier transform infrared spectrometer (Thermo Nicolet NEXUS, Thermo Nicolet Corp., Madison, WI, USA) over 32 scans. Pb(SCN)_2_ was the internal standard (2060 cm^−1^) used to measure the molecular vibration spectra under various experimental conditions. The measured samples were first mixed with the same amount of Pb(SCN)_2_ powder and pressed into KBr pellets after being ground. The final spectra ranged from 4000 to 400 cm^−1^. Zeta potentials were measured using a Malvern Zen 3600 Zetasizer. A small amount of sample was first dispersed into ethanol and a drop of the mixture was transferred onto a cover slip to investigate its morphology, where the scanning electron microscope (SEM) (Quanta FEG 250, FEI Company, Hillsboro, OR, USA) was run at 10.0 kV. A Bruker D8 X-ray powder diffractometer with Cu Kα as the radiation source was employed for the XRD spectrum of the sample, spanning a 2θ range from 10.0 to 70.0° at a scanning rate of 0.0200° min^−1^. The accelerating voltage and the electric current used herein were 40.0 kV and 40.0 mA, respectively.

## 4. Conclusions

In conclusion, the formaldehyde/melamine molar ratio of 1.5:1 is an important value in the synthesis of melamine resin microspheres, which allowing us to classify characteristics of the microspheres, as well as the performance of the loaded silver nanoparticles. It is worth highlighting that the excess formaldehyde on this molar ratio in the synthesis reaction can be used to tune adsorption of the silver nanoparticles that are loaded on the microspheres, thereby leading to an intense SERS enhancement on them. Modifying resin microspheres with the excess monomer in the synthesis enables us to conveniently prepare new SERS substrates and, thus, to detect some environmental and food contaminants, especially e.g., various organic amine salts.

## Supplementary Materials

The following are available online at http://www.mdpi.com/2079-4991/7/9/263/s1. Figure S1: XRD analysis of AgNPs loaded on a set of MFMSs; Figure S2: Terminal overreaction between the –NH_2_ around a two-dimensional resin molecule and the excessive CH_2_O remained in the reaction solution; Figure S3: Severe aggregation of AgNPs loaded on MF_1_; Figure S4: SERS spectra from R6G molecules in different concentrations on AgNPs loaded on MF_3_ synthesized in 8 h; Figure S5: Comparison between the 610 cm^−1^ Raman and SERS on AgNP/MFMSs bands of R6G; Figure S6: R6G SERS spectra collected on a set of randomly-selected AgNP/MF_7_MSs; Figures S7 and S8: Optical images of MFMSs and AgNP/MFMSs under a confocal Raman spectroscope; Figures S9 and S10: SERS spectra from R6G on AgNPs loaded on MFMSs using 532 nm and 785 nm laser, respectively; Table S1: Particle sizes of MFMSs synthesized under a set of M:F molar ratios; Table S2: Silver contents in a set of AgNP/MFMSs samples; Table S3: RSD values of a set of SERS peak intensities from Figure S6.
