# Supplementary material for: Optimizing Melamine Resin Microspheres with Excess Formaldehyde for the SERS Substrate
Source: Nanomaterials (Basel). 2017 Sep 6;7(9):263. doi: 10.3390/nano7090263 (PMC5618374; doi:10.3390/nano7090263)
Supplement: Supplementary file 1 [file nanomaterials-07-00263-s001.pdf]

Optimizing Melamine Resin Microspheres with Excess Formaldehyde for the SERS Substrate

Lu Shen, Junfu Zhu, Yuqing Guo, Zhirong Zhu, Xiaogang Wang and Zhixian Hao*

Shanghai Key Laboratory of Chemical Assessment and Sustainability, School of Chemical Science and Engineering, Tongji University, Shanghai 200092, Shanghai (P. R. China).

Financial support from the National Science and Technology Major Project from Ministry of Science and Technology of the People's Republic of China (2012YQ1502130202).

*E-mail: haozhixian@tongji.edu.cn

Particle Size of MFMSs Changed with the M:F Molar Ratio Taken in Their Synthesis Reaction

Table S1. Particle sizes of MFMSs synthesized under a set of M:F molar ratios.

M:F molar ratio	1:1	1:3	1:7	1:9
particle size (μm)	12.43	8.42	8.33	5.82
SD (μm)	2.21	1.02	0.80	0.47

The particle size and standard deviation (SD) were from the statistics of the diameter values of the MFMS images in the SEM photo of the sample and, wherein, the number of the counted MFMSs was more than 80.

XRD Analysis of AgNPs Loaded on a Set of MFMSs.

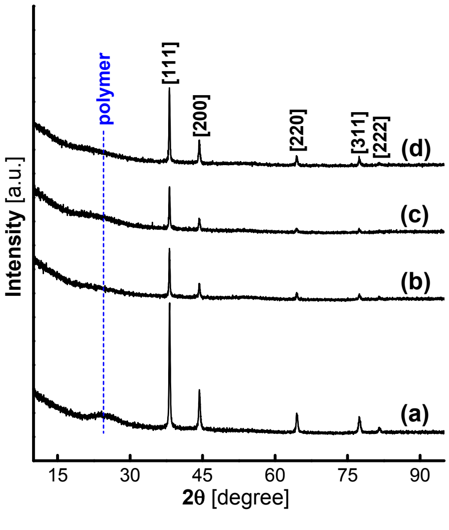

Figure S1. XRD analysis of AgNPs loaded on a set of MFMSs. (**a**) MF_1; (**b**) MF_3; (**c**) MF_7; and (**d**) MF_9. The pure fcc crystal structure of the AgNPs is evident, while a declining diffraction peak from the semi-crystalline MFMSs is observed.

The Diagram for Terminal Overreaction

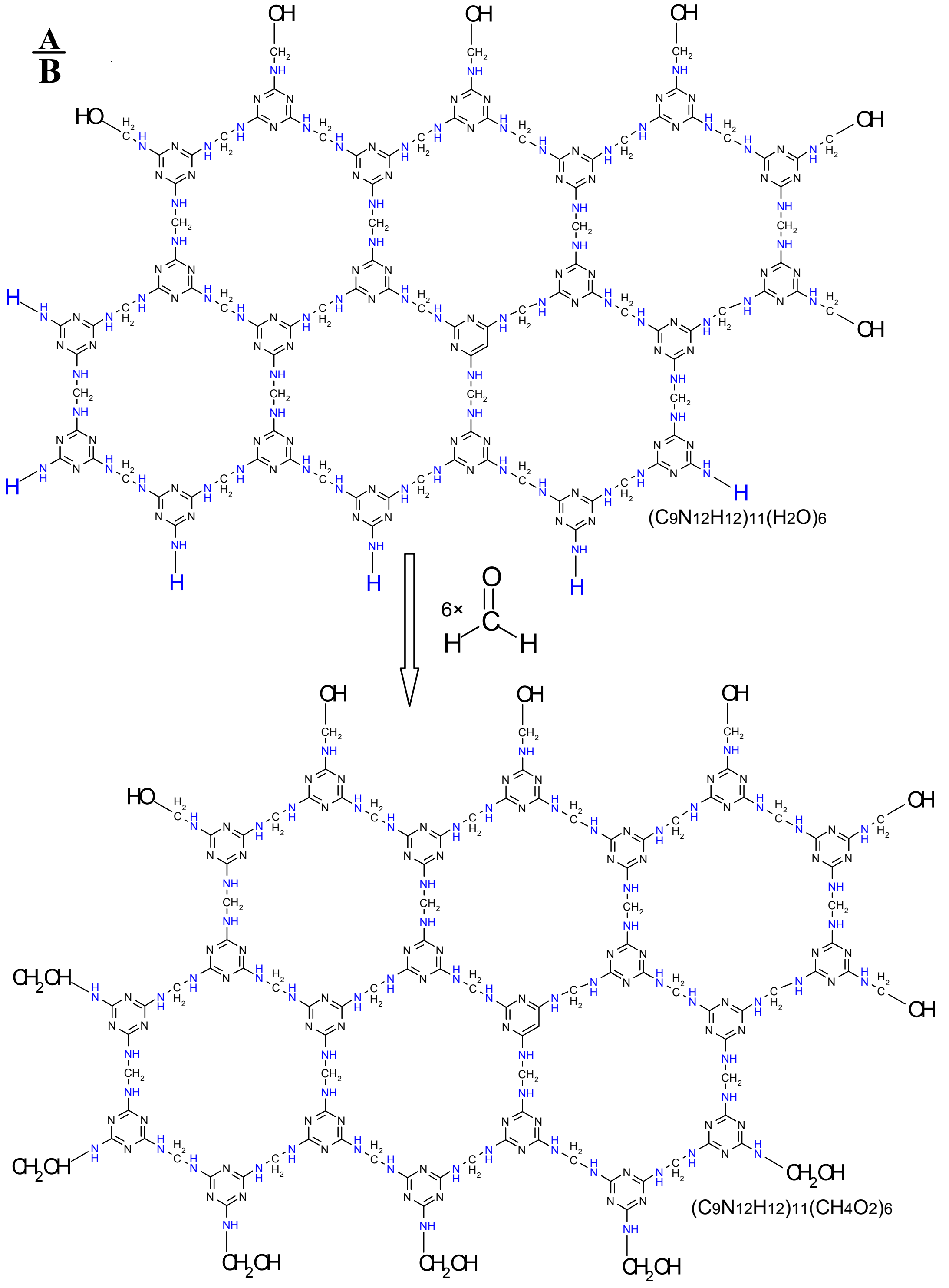

Figure S2. Terminal overreaction between the –NH_2 around a two-dimensional resin molecule and the excessive CH_2O remained in the reaction solution. (**A**) A two-dimensional resin molecule posed from the unit $C_9N_{12}H_{12}$; (**B**) The resin molecule with -NH_2 groups further bonded. All the –NH_2 groups around the resin molecule were transferred into –$NHCH_2OH$ after the terminal overreaction with the excessive formaldehyde.

Measurement of Silver Content in AgNP/MFMSs

A 0.125 g measure of MFMSs (synthesized in 3h) was impregnated in 50 mL AgNP solution for 60 min and separated at 2000 rpm. The obtained AgNP/MFMSs were washed with water for three times and dried in an oven at 80 °C for 4 h. Accurately-weighed dry AgNP/MFMSs (weight: m_1) were moved into a ceramic crucible (weight: m_2) (before weighing, ceramic crucibles were dried in an oven at 200 °C, then kept in a desiccator.). The samples were then put into a muffle stove, heated to 750 °C and kept at that temperature for 2 h. Only a white metal silver remained in the ceramic crucible following this treatment. The samples were then taken out at ~200 °C and quickly put into a desiccator to cool down. They were finally weighed (weight: m_3), to complete the whole measurement procedure. The content of silver loaded on MFMSs was calculated by the formula: $(m_3 – m_2) / m_1$.

Table S2. Silver contents in a set of AgNP/MFMSs samples.

AgNP/MFMSs	AgNP/MF$_1$MSs	AgNP/MF$_3$MSs	AgNP/MF$_7$MSs	AgNP/MF$_9$MSs
Ag load	5.82%	6.42%	5.02%	6.23%

SEM Image of Severe Aggregation of AgNPs Loaded on MF$_1$

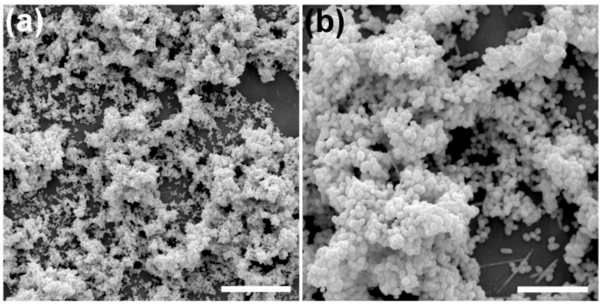

Figure S3. Severe aggregation of AgNPs loaded on MF$_1$. Scale bars are 3 μm (**a**) and 1 μm (**b**), respectively.

Detection Limit and SERS Enhancement Factor

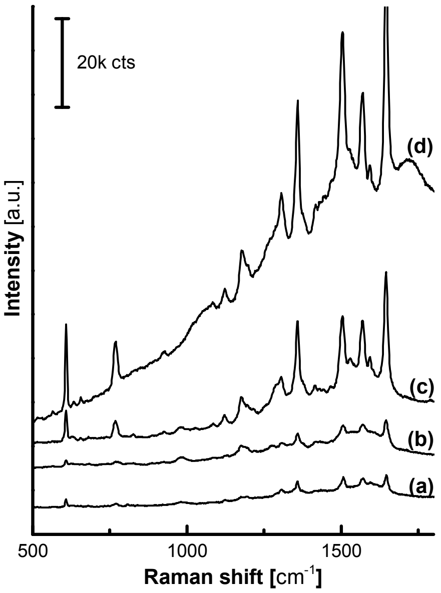

Figure S4. SERS spectra from R6G molecules in different concentrations on AgNPs loaded on MF_3 synthesized in 8h. (**a**) 10^{-13}M; (**b**) 10^{-11}M; (**c**) 10^{-9}M; and (**d**) 10^{-7}M. The laser wavelength was 514.5 nm, the irradiation power was 0.2 mW, and the data acquisition time was 10 s.

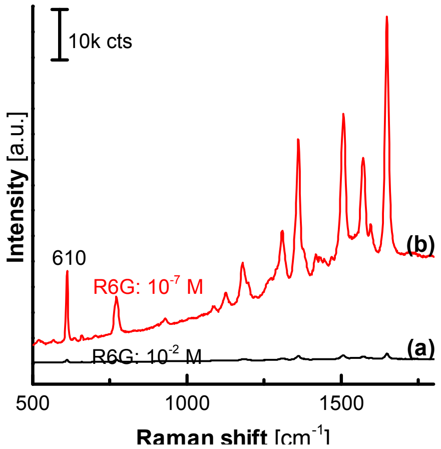

Figure S5. The comparison between the 610 cm^{-1} Raman (**a**) and SERS on AgNP/MFMSs (**b**) bands of R6G. The laser wavelength was 514.5 nm, the irradiation power were, respectively, 10 mW (a) and 0.2 mW (b), and the data acquisition time was 10 s.

In our case, the SERS enhancement factor (EF) was roughly calculated by the following equation: EF = $I_{SERS}C_{NR} / I_{NR}C_{SERS}$, where I_{SERS} and I_{NR} are the corresponding SERS intensity of R6G on AgNP/MFMSs substrates and the normal Raman (NR) intensity of R6G solution for the 610 cm^{-1} band; C_{SERS} and C_{NR} are concentrations of the R6G molecules in the SERS and reference samples (10^{-2} M R6G on the glass slide). The laser power of 0.2 mV (1% of 20 mV) and 10 mV (50% of 20 mV, characteristic signals did not appear at 0.2 mV for the 10^{-2} M R6G

solution) were used in the test of I_{SERS} and I_{NR}, respectively. The Raman intensity is in direct proportion to the laser power so I_{NR} measured at the same laser power as I_{SERS} can be estimated. In our work, the EF is estimated to be about 1.24×10^8.

Homogeneity of the AgNP/MFMSs as a SERS Substrate

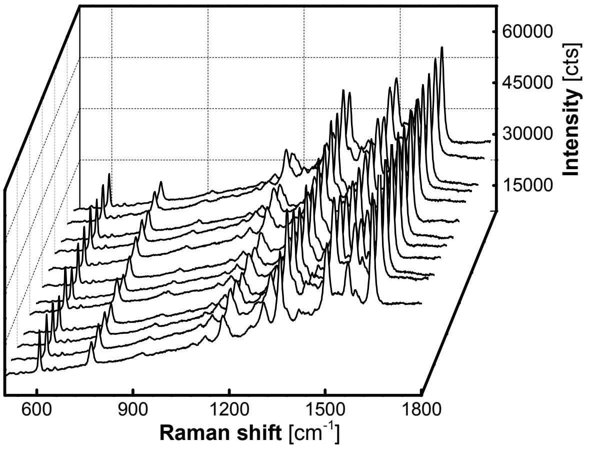

Figure S6. R6G SERS spectra collected on a set of randomly-selected AgNP/MF_7MSs. The concentration of R6G in the incubation solution was equal to 10^{-7} M, the laser wavelength was 514.5 nm, the irradiation power was 0.2 mW, and the data acquisition time was 10 s. The SERS spectra were collected by focusing the laser beam on each single microsphere through a 50 × microscope objective.

Table S3. RSD values of a set of SERS peak intensities from Figure S6.

Peak (cm^{-1})	1646	1508	1364	1185	773	608
RSD (%)	10.2	7.1	7.0	9.5	8.6	14.8

SERS peak intensities detected on the randomly selected AgNP/MF_7Ss presented relative standard deviation (RSD) values less than 15% (Table S3), demonstrating the good homogeneity of the MFMSs used for the SERS substrate.

Optical Images of MFMSs and AgNP/MFMSs under a Confocal Raman Spectroscope

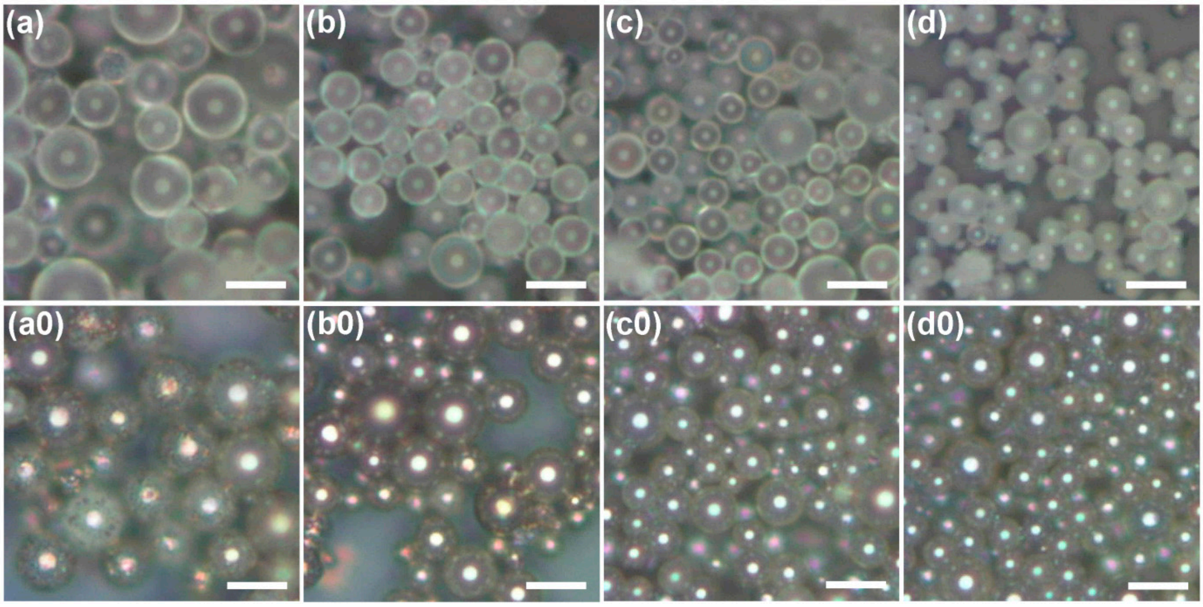

Figure S7. Optical images of MFMSs with a set of F/M molar ratios synthesized in 3 h under a confocal Raman spectroscope. (**a**) MF_1; (**b**) MF_3; (**c**) MF_7; and (**d**) MF_9. Optical images of AgNP/MFMSs with a set of F/M molar ratios synthesized in 3 h under a confocal Raman spectroscope. (**a0**) AgNP/MF_1MSs; (**b0**) AgNP/MF_3MSs; (**c0**) AgNP/MF_7MSs; and (**d0**) AgNP/MF_9MSs. Scale bars are 10 μm.

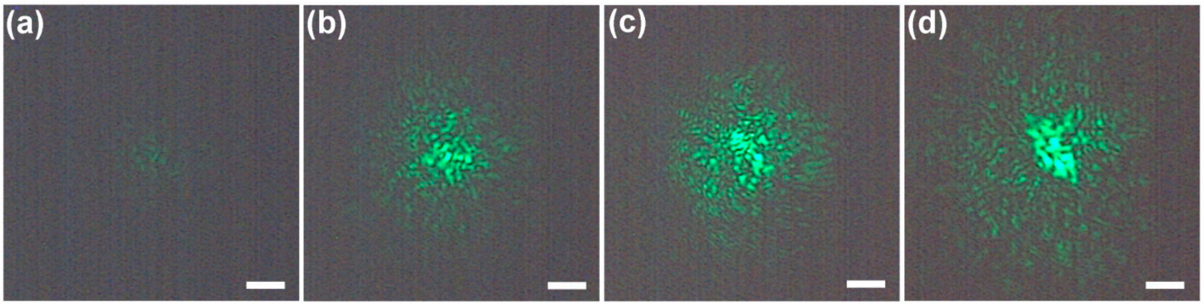

Figure S8. Optical images of AgNP/MFMSs excited with a laser power of 0.2 mV (**a**), 2 mV (**b**), 10 mV (**c**), and 20 mV (**d**). Scale bars are 20 μm.

In our experiment, 0.2 mV (only 1.0% of 20 mV) was used to measure SERS spectra, where the image signal was too weak to distinguish the characteristics of the samples.

SERS Spectra from R6G on AgNPs Loaded on MFMSs Using 532 nm and 785 nm Lasers

In addition to 514.5 nm laser, the wavelengths of 532 nm and 785 nm in another confocal microscope Raman spectrometer (Bruker-Senterra, Bruker Corporation, Billerica, MA, USA) were used for further measurement of SERS characteristics.

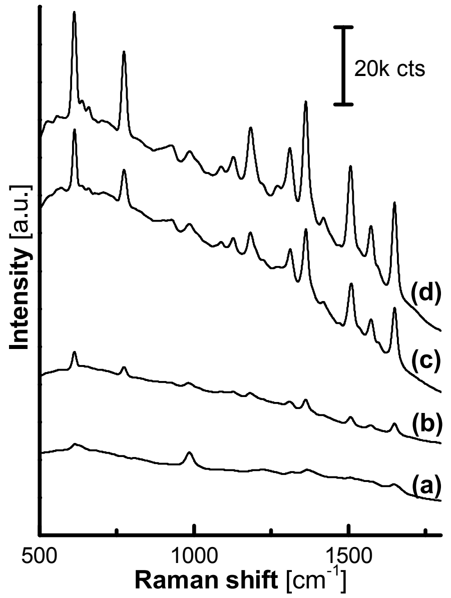

Figure S9. SERS spectra from R6G on AgNP/MFMSs synthesized with a set of F/M molar ratios in 3 h. (**a**) MF_1; (**b**) MF_3; (**c**) MF_7; and (**d**) MF_9. The laser wavelength was 532 nm, the irradiation power was 0.2 mW, and the data acquisition time was 10 s.

As presented from Figure S9a–d, the intensity of the SERS signals from R6G were gradually enhanced with the F/M molar ratio increasing from 1:1 to 9:1, which was consistent with the feature from the laser power of 514.5 nm.

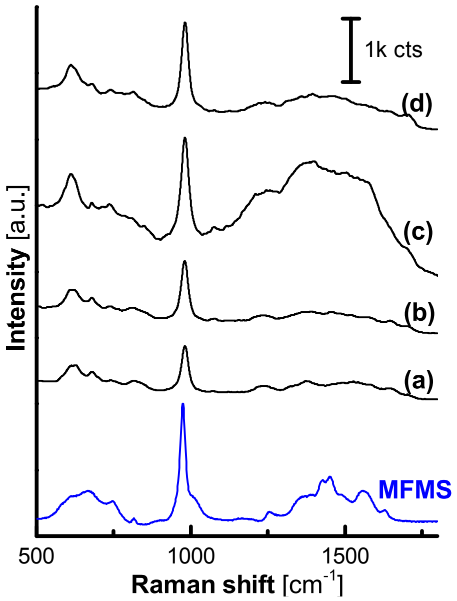

Figure S10. Raman spectra of MF_3 resin (blue) and SERS spectra from R6G on AgNP/MFMSs samples synthesized with a set of F/M molar ratios in 3 h. (**a**) MF_1; (**b**) MF_3; (**c**) MF_7; (**d**) MF_9. The laser wavelength was 785 nm, the irradiation power was 1 mW, and the data acquisition time was 10 s.

No characteristic signals from R6G were observed using the laser with the wavelength of 785 nm. Compared with the normal Raman spectra of MF resin, it was found that the obtained signals were from MF resin instead of R6G, this is to say, surface plasmons were not excited using the wavelength of 785 nm.
